# Impact of Minimally Invasive Intra-Capsular Metatarsal Osteotomy on Plantar Pressure Decrease: A Cross-Sectional Study

**DOI:** 10.3390/jcm13082180

**Published:** 2024-04-10

**Authors:** Carlos Fernández-Vizcaino, Carmen Naranjo-Ruiz, Nadia Fernández-Ehrling, Sergio García-Vicente, Eduardo Nieto-García, Javier Ferrer-Torregrosa

**Affiliations:** 1 Podiatry Department, Faculty of Medicine and Health Sciences, Valencia Catholic University “San Vicente Mártir”, 46001 Valencia, Spain; carlos.fernandez@ucv.es (C.F.-V.); carmen.naranjo@ucv.es (C.N.-R.); nadia.fernandez@ucv.es (N.F.-E.); eduardo.nieto@ucv.es (E.N.-G.); 2 Admission and Clinical Documentation Unit, Sagunto Hospital, Generalitat Valenciana, Universidad Europea, 46001 Valencia, Spain; sergiogvi@comv.es

**Keywords:** metatarsalgia, osteotomy, minimally invasive, inclinometer, plantar pressures, DICMO

## Abstract

**Background:** Metatarsalgia is a common pathology that is initially treated conservatively, but failure to do so requires surgery, such as the minimally invasive distal metatarsal osteotomy (DICMO). **Methods:** In this prospective study of 65 patients with primary metatarsalgia who underwent DICMO, plantar pressures, American Orthopaedic Foot and Ankle Society MetaTarsoPhalangeal-InterPhalangeal scale (AOFAS-MTP-IP) and Visual Analog Scale (VAS) were evaluated pre-operatively and post-operatively and there was a subgroup in which an inclinometer was used to observe the importance of the inclination of the osteotomy. **Results:** The results show a significant reduction in plantar pressures after DICMO surgery without overloading the adjacent radii, especially in the subgroup with an inclinometer to guide the osteotomy. The AOFAS-MTP-IP scale evidenced a marked improvement in metatarsal function and alignment with scores close to normal. The VAS scale showed a substantial decrease in pain after DICMO osteotomy. **Conclusions:** DICMO, with an inclinometer for a 45° osteotomy, proved to be a safe and effective procedure for primary metatarsalgia, although further comparative studies are needed to confirm its superiority.

## 1. Introduction

Metatarsalgia is a common pathology in podiatric clinical practice and is defined as localized pain in the plantar region of the metatarsal heads, especially in the second, third, and fourth central metatarsals. Its prevalence in the general population is estimated to be between 5% and 10%. The etiology of metatarsalgia is multifactorial. Biomechanical causes are the most frequent and include alterations such as discrepancy in length, excessive plantarflexion, and pes cavus that causes early support of the metatarsal heads, insufficiency of the first radius with hypermobility, and clubfoot that limits ankle dorsiflexion.

The diagnosis of metatarsalgia is made by anamnesis, physical examination, radiographic study and an analysis of gait and plantar pressure distribution. This allows the underlying cause to be determined and an appropriate treatment plan to be established.

Initial treatment is usually conservative, including measures such as callus delamination, footwear modifications, plantar supports with metatarsal offloading, infiltrations, and physical therapy. If after 3–6 months these measures fail, surgical treatment is indicated.

Surgical techniques for metatarsalgia can be open or minimally invasive. Weil’s osteotomy is the most commonly used in open surgery, with a shortening–elevating effect. In minimally invasive surgery, there are two options: the distal metaphyseal osteotomy (DMMO) [1,2], which performs an extracapsular cut and the distal intracapsular metatarsal osteotomy (DICMO), which is performed on the surgical neck of the metatarsal remaining intracapsular [3,4,5].

Minimally invasive foot surgery (MIS), performed through small incisions, has gained popularity since the 1940s, standing out for its ability to correct alterations and deformities with less soft tissue damage, reducing post-operative pain, thereby enhancing patient comfort and satisfaction, while concurrently improving clinical outcomes and promoting faster recovery [6,7,8]. Minimally invasive distal metatarsal osteotomy (DMMO) is a surgical technique that has become popular in recent years in the treatment of metatarsalgia [9]. It consists of making a bony cut at the level of the metaphysis of the affected metatarsal through a small lateral incision without a direct soft tissue approach [9]. This shortening or dorsiflexing osteotomy [5,10] aims to redistribute plantar pressures by modifying the biomechanics of the forefoot. However, evidence on its concrete effects is still scarce. This surgery may contribute to decreasing plantar pressures through a metatarsal shortening caused by the bony resection that shortens the length of the metatarsal, avoiding early support of the head during gait. The design of the osteotomy, with a typical angulation of 45° [11,12], creates a dorsal displacement of the metatarsal head by elevating it. The metatarsal realignment allows for the correction of plantarflexed metatarsals [13,14] and, if we perform it intracapsularly in the metatarsal head, we can maintain the alignment by avoiding excessive dorsiflexions, as demonstrated by Laffenêtre et al. [15,16], who have named this osteotomy distal intra-capsular minimally invasive osteotomy (DICMO) [17,18].

Few studies have evaluated the effect of plantar pressures, one of the most current ones being a study which analyzes plantar pressures in metatarsal and hallux surgery [19,20,21]. Research with percutaneous surgery is required to confirm that the described biomechanical changes translate into a significant and clinically relevant reduction of plantar pressures after surgery compared to the pre-surgical state [22]. Furthermore, the execution of the osteotomy within the capsular space, with minimal separation of the periarticular soft tissues, provides stability and limitations to metatarsal head deviation once the procedure is performed due to the reflex contracture of the capsule and the surrounding soft tissue. The DD-PP angulation, with respect to the diaphyseal axis of the metatarsal that is used in this type of osteotomy, allows for an adequate confrontation of the bony surfaces promoting the creation of bony bridges and the decrease in movement of the fragments, avoiding complications in this type of surgery.

The primary objective of this study is to prospectively evaluate the clinical and biomechanical efficacy of DICMO surgical treatment in patients with primary metatarsalgia. The secondary objective is to compare the utilization of a goniometer to position the surgical motor at 45 degrees between two study groups. Both objectives will be assessed utilizing the visual analog scale, the American Orthopaedic Foot and Ankle Society MetaTarsoPhalangeal-InterPhalangeal scale (AOFAS-MTP-IP), and plantar pressures at 6 and 12 months post-treatment.

## 2. Materials and Methods

### 2.1. Study Design

This prospective multicenter study was conducted with patients diagnosed with primary metatarsalgia, consecutively scheduled for unilateral DICMO surgery. This study was approved by the Research Ethics Committee of Catholic University of Valencia (UCV/2022-2023/094) in accordance with the ethical guidelines of the Declaration of Helsinki [23]. In addition, the design and progression of participants through the trial were conducted in accordance with the STROBE guidelines [24]. All participants were recruited from two clinics and operated on by the same surgical team. Before surgery procedures, all participants provided their written informed consent [23,24].

### 2.2. Sample Size

For the primary objective, a pre-hoc power analysis was conducted using G-power 3.1 statistical software. The required sample size was estimated using values of error probability, Cohen’s effect size (dz: 0.5), significance level (α: 0.05), and statistical power (1-β: 0.8). The initial calculated minimum sample size was 50.

For the secondary objective, sample calculation was performed to compare the means of two normal populations with paired data. The expected variances for the two populations are 2 and 2, respectively. The predicted Pearson’s linear correlation coefficient is 0.95. The predicted population variance inferred for the differences is 0.2. A significance level of 5% was employed, and a power of 98% was desired to detect a difference of 0.5 units. A 10% loss of pairs was estimated, requiring at least 15 observations in each sample, totaling 30 observations. The expected half-width of the Confidence Interval is 1.10756 units.

### 2.3. Participants

**Figure 1:**
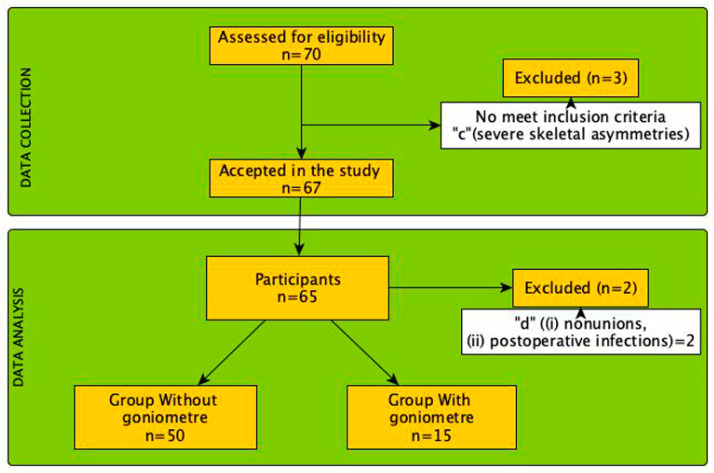
Flow diagram of the selection process and analysis of the participants included in the present study.

A total of 65 consecutive patients were selected from two clinics. The sample (Figure 1) consisted of 65 patients (74% female, n = 48, with a mean age of 54.18 ± 14.37 and 26% male, n = 17 with an age of 54.69 ± 13.75. The mean body mass index (BMI) was 24.85 ± 3.60. Table 1 presents more demographic data of our study group and Figure 1 shows the flow chart of the research.

Inclusion criteria: (a) patients older than 18 years-old; (b) diagnosis with primary metatarsalgia; (c) no alterations in digital alignment; and (d) posture neutral FPI. Exclusion criteria: (a) existing neurological deficits that could influence gait or standing patterns; (b) previous orthopedic surgery on the lower limb and/or foot; (c) several skeletal asymmetries; (d) major intra and post-operative surgical complications, including (i) non-unions, (ii) post-operative infections; (e) any other condition during inclusion in the protocol period that has an impact on gait or standing patterns, such as trauma to the lower extremities and/or spine or neurological conditions. Each participant underwent a pre-operative evaluation, static baropodometric measurements, and AOFAS-MTP-IP and VAS assessment. This evaluation was repeated at 6 months and at 12 months post-operatively, respectively.

### 2.4. Surgical Technique—Minimally Invasive Intracapsular Metaphyseal Osteotomy (DICMO)

All patients are operated on utilizing an outpatient basis, under local anesthesia using an anesthetic ankle block with mepivacaine 20 mg/mL, with a maximum of 10–12 mL in the ankle block and 4–6 mL in the intermetatarsal block.

**Figure 2:**
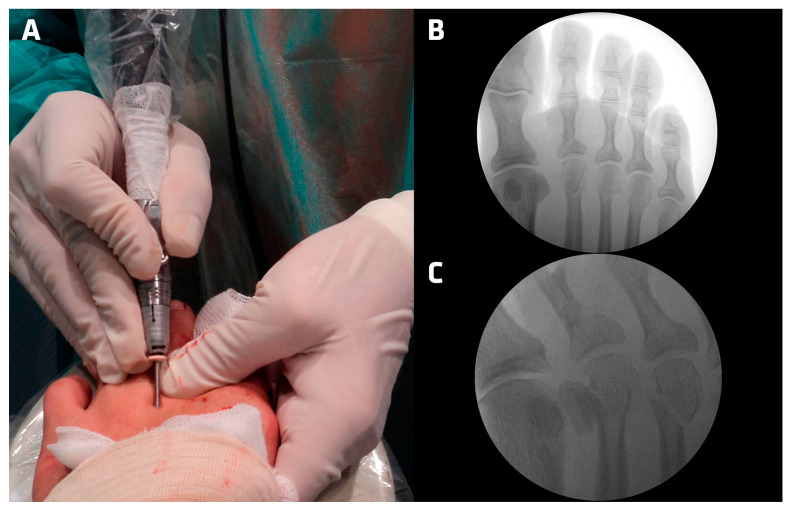
Metatarsal surgery (DICMO) (**A**) Motor inclination (**B**) Dorsoplantar Fluoroscopy 2nd and 3rd metatarsal (**C**) Oblique Fluoroscopy 3rd metatarsal.

1. Locating the metatarsal head: The passive hand, using the index finger and thumb, is in charge of locating the metatarsal head at the dorsal and plantar level and fixing its position to avoid its displacement;

2. The dorsal incision is made over the center of the metatarsal head, parallel to the extensor tendons of the toes. The skin is incised and the incision is deepened crossing the planes until reaching the joint capsule, which is incised reaching the metatarsal head;

3. The Beaver 64 scalpel blade is slid at a 45° angle to the diaphyseal axis of the metatarsal in a proximal and plantar direction until it reaches the bony cortex of the metatarsal neck;

4. At the intracapsular location, the periosteum is lifted and the cortex of the metatarsal neck is lightly scraped to avoid displacement of the drill at the start of the cut;

5. The Shannon Isham straight flute (Vilex, TN, USA) reamer is introduced into the incision made, and placed into the notch made in the metatarsal neck with an inclination of 45° in relation to the diaphyseal axis of the metatarsal. We checked the correct location and angulation with the fluoroscope in 15 cases (Figure 2);

6. The osteotomy is started on the lateral aspect of the metatarsal neck, maintaining the 45° angulation, intracapsularly, until approximately one third of the metatarsal width is cut;

7. At this point the osteotomy is finished, the direction of the cut is moved from lateral to dorsal, placing the drill in the plantar cortex and with the drill positioned perpendicular to the diaphyseal axis of the metatarsal and parallel to the metatarsal articular facet, following the line of cut that was previously made.

8. Once the cut of the dorsal cortex is finished, the drill is removed and the execution of the osteotomy is checked. The corresponding fingers are pulled distally to verify the complete cut of all the cortices of the metatarsal neck.

9. When minimally invasive surgery is performed without ischemia, bleeding after the osteotomy occurs, which drags the bony debris out of the incision. Even so, the bone dust is removed as a result of the cut by compression from proximal to distal.

10. The incision is closed with a single discontinuous stitch with 4/0 monofilament nylon. After the operation, the bandage, using 2.5 cm wide and 10 cm long crossed strips of Hypafix^®^ nonwoven tape (BSN medical GmbH, Hamburg, Germany), is applied over the metatarsal head to maintain the fixation of the osteotomy. Cross strips are placed on the dorsal side and adhered in the plantar area with parallel strips as many layers of bandage are placed as necessary to maintain fixation.

The patient is allowed to ambulate in a stiff-soled, rocker post-surgical shoe that provides stability and reduces shock and pressure to the surgical site (MedSurg model from Darco International, Huntington, WV, USA).

All the complications found, such as displacement, non-union, delayed consolidation, hypertrophic callus, and no correction, were registered.

### 2.5. Digital Goniometer Subgroup

**Figure 3:**
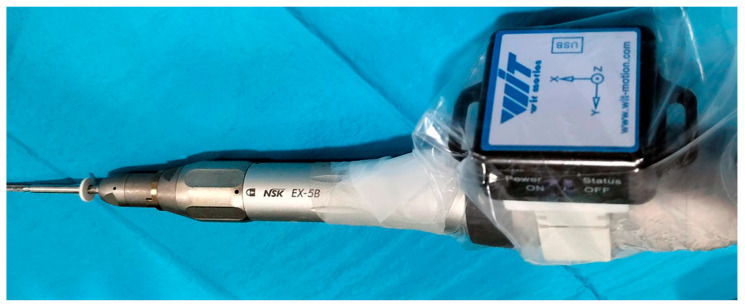
A goniometer incorporated to the surgical micromotor sends the data to a mobile phone via Bluetooth.

In 15 patients the same surgery was performed, but using a goniometer to perform the osteotomy at the same 45° angulation [25] (Figure 3).

### 2.6. Assessment Baropodometric Study

**Figure 4:**
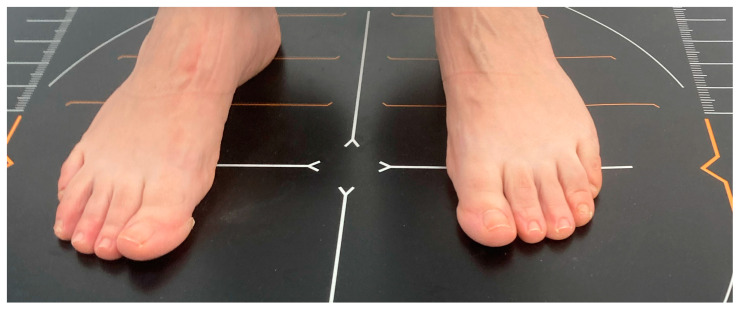
A Static plantar pressure analysis using Medicapteurs^®^ S-Plate (Balma, France).

For the measurement of plantar pressures, a Medicapteurs^®^ (Balma, France) S-Plate pressure platform is used, which is a podobarographic system that studies the pressures of the foot statically (Figure 4). It collects information by means of sensors that detect the force applied by each support zone of the foot, and information is presented by means of a computer software for Windows provided by the company Medicapteurs, generating an image where the different pressures are converted, through colorimetry, into a map and the pressures in different areas in the forefoot, midfoot and rearfoot appear in different boxes, the point of maximum pressure and, by choosing a specific point with the cursor, the pressure in g/cm^2^ of the chosen zone.

Prior to performing the podobarographic analysis, patient data are entered into the computer program, including the weight and height of the subjects, in order for the computer program to obtain the plantar pressure values in each of the detection zones. These values are obtained with the GIMA (Milan, Italy) Pegaso ^®^ class III digital scale and measuring rod. In order to perform the static analysis of plantar pressures, the patients were barefoot, and detailed information was provided to all participants about the procedure, asking them to adopt a normal, relaxed bipedal position on the platform. The static image was acquired after 20 s in a comfortable posture of the patient.

### 2.7. Assessment AOFAS-MTP-IP and VAS

The AOFAS-MTP-IP scale is an assessment tool used to measure function and clinical outcome in patients with foot and ankle problems. This scale addresses several aspects, including pain, function, alignment, and joint mobility. The total score varies according to patient responses and clinical assessment, thus providing an overall measure of function and well-being related to the condition under study. The scale is widely used in orthopedic practice and helps to standardize the assessment of outcomes in patients with foot and ankle conditions [26]. For the purpose of this study, the lesser metatarsophalangeal-interphalangeal subscale was analyzed [27].

The scale was obtained at three times: pre-surgical, 6 months, and 12 months post-surgery, respectively.

The VAS scale stands out as the tool of choice in pain assessment, thanks to its simplicity and wide use in clinical practice. We will adopt a numerical scale ranging from 0 to 10 for this measurement. This technique provides an effective way to subjectively quantify the range of pain, where 0 indicates the total absence of pain and 10 represents the maximum conceivable pain. It is, therefore, considered superior to descriptive or fixed-value scales. Likewise, the VAS scale has been shown to have a high reliability (α Conbrach = 0.97) [95% CI = 0.96 to 0.98] [28].

### 2.8. Statistical Analysis

An observer who was unaware of the experimental setup performed all analyses. The mean and standard deviation (SD) were used to express the data. The Kolmogorov–Smirnov test was used to evaluate the assumption of normality. Levene’s test was also used to calculate the homogeneity of variance assumption. At *p* > 0.05, the significance level was established. For the statistical analysis and graphical display of the data, SPSS 24 (SPSS 24 Inc., Chicago, IL, USA) and Jeffreys’ Amazing Statistical Package (JASP V0.16.4, Amsterdam, Netherlands) were used. In order to determine whether the anthropometric characteristics among the groups were homogeneous (*p* > 0.05), a one-way *t*-Test was used to examine the data.

A repeated measures ANOVA was performed to compare the effects of the plantar pressures, AOFAS-MTP-IP, and VAS variables. Mauchly’s test of sphericity was used to evaluate the assumption of sphericity of the within-subject factor data. Post hoc comparisons were performed with Bonferroni adjustment.

The ES was calculated by determining Cohen’s d coefficient, which was then expressed as the difference of standardized mean change. The ES was categorized as trivial (<0.20), small (0.20–0.59), moderate (0.60–1.19), large (1.20–1.99), or very large (>2.00) [29].

## 3. Results

Post-operative complications were observed. One case of nonunion and one case of post-operative infection required additional surgical procedures. Minor complications included delayed union in 6 patients, which resolved within 6 months post-operatively. Pseudarthrosis occurred in several cases. At the 6-month follow-up stage, 20 patients manifested mild residual pain but were ambulating normally, while 15 patients had residual post-operative edema. Importantly, no cases of transfer metatarsalgia or recurrence of the original pathology were observed at either the 6 or 12 months follow-up stage.

### 3.1. Plantar Pressures

**Figure 5:**
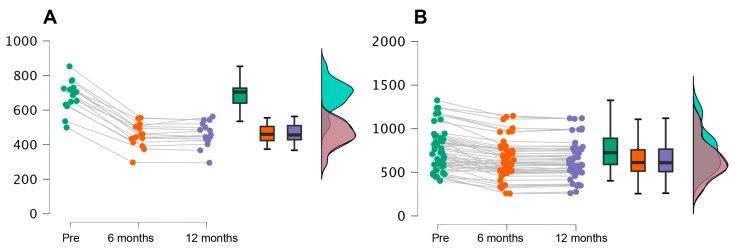
Decrease in plantar pressures in each of the groups with and without goniometer. (**A**) Without goniometer, (**B**) With goniometer.

Table 2 shows a significant decrease in plantar pressures between the baseline and 6 months post-treatment, with a mean pressure reduction from 747.63 g/cm to 601.86 g/cm. It is encouraging to note that this reduction remains relatively constant after 12 months, with a mean pressure of 602.83 g/cm. This finding suggests that the treatment applied has achieved an effective decrease in plantar pressures and that this effect is maintained over time, indicating the long-term durability and efficacy of the treatment in improving plantar load distribution. In addition, in response to the secondary objective of comparing the use of the goniometer to ensure the angulation of the same, we observed that the analysis showed that plantar pressures differed significantly: F (1, 63) = 7, *p* < 0.01, w^2^ = 0.11. The Greenhouse–Geisser correction was used, since Mauchly’s test of sphericity was significant (Figure 5).

Post hoc analysis using Bonferroni correction revealed that plantar pressure decreased significantly as time passed between measurements at 6 months and 12 months (mean difference = 145.77, *p* < 0.001) and between 6 months and 12 months (mean difference = 144.80 units, *p* = 0.001). No significant differences were found between 6 months and 12 months. Observing the effect size in the post hoc contrast, we highlight a Cohen’s D of 7.51 in the pre-6 months pair and 0.33 in the pre-12 months pair. If we compare these plantar pressures by dividing the two groups of the study into those who used a goniometer and those who did not, we observe the following data.

### 3.2. AOFAS-MTP-IP Results

The descriptive data of the AOFAS-MTP-IP variable at the three evaluation times were prior to surgery, and the total sample of 65 participants showed a mean score of 43.75 ± 15.17 (mean ± standard deviation). This baseline score reflects a severely impaired functional status of the ankle and foot. The coefficient of variation of 0.35 indicates considerable dispersion of individual values from the pre-surgical mean.

At 6 months post-surgery, a marked clinical improvement was observed, with a mean score of 86.37 ± 8.98. This difference from the baseline suggests significant progress in ankle and foot function in this follow-up period. In addition, the lower coefficient of variation of 0.10 evidences a lower variability of individual scores around the mean.

At the 12-month assessment, the mean score was 92.77 ± 9.24, demonstrating further and sustained improvement over the previous period. As at 6 months, the coefficient of variation of 0.10 indicates a more homogeneous distribution of values compared to the pre-surgical state. It should be noted that the narrow standard errors of the mean at the three time points (1.88, 1.11, and 1.15, respectively) suggest an adequate precision of the sample estimates.

If we analyze the two subgroups of our second objective, with the goniometer and without goniometer, the results of a repeated measures analysis of variance for the dependent variable AOFAS-MTP-IP. Using the Greenhouse-Geisser correction for non-sphericity, an F = 441.12, *p* < 0.001 was obtained. The effect size was large (ω^2^ = 0.78). There are significant differences in AOFAS-MTP-IP score between the group with goniometer (With) and without goniometer (without) at all time points (*p* < 0.001). The goniometer group always had higher AOFAS-MTP-IP scores. The greatest differences occurred at 6 and 12 months. At 6 months, the difference was 42.96 points and at 12 months the difference was 49.00 points (in favor of the goniometer group). The effect size (Cohen’s d) was also large at these times, suggesting that the effect of the goniometer on the AOFAS-MTP-IP score is clinically relevant. No significant differences were found between different time points within each group (e.g., between 6 and 12 months in the SI group), suggesting that the effect of the goniometer remains stable over time. As shown in Table 3, acccording to the results of this table, there are significant differences in the AOFAS-MTP-IP score when using the goniometer compared to not using it.

Pre-operatively, the goniometer group (SI) had an AOFAS-MTP-IP score 2.31 points higher than the non-goniometer group (NO), and this difference in number was statistically significant (*p* = 0.98). At 6 months, the difference between the YES and NO groups was 42.96 points (*p* < 0.001). At 12 months, the difference was 49.00 points (*p* < 0.001). That is, at the time all points were evaluated, goniometer use was associated with significantly higher AOFAS-MTP-IP scores compared to when it was not used. Moreover, the differences were clinically relevant, with medium to large effect sizes. Therefore, according to this AOFAS-MTP-IP displayed in Table 3, there is strong evidence that the use of the goniometer makes a significant and clinically important difference in the functional outcomes measured by AOFAS-MTP-IP.

According to the results of this table, there are significant differences in the AOFAS-MTP-IP score when using the goniometer compared to not using it.

### 3.3. VAS Variable

The group VAS scale scores increased considerably from the pre-surgical value of 43.52 to 86.63 at 6 months and 93.26 at 12 months. In addition, the standard deviation and coefficient of variation decreased in the later periods, indicating a smaller dispersion of the individual values with respect to the mean. And, when analyzed from a subgroup perspective, a significant main effect of VAS is identified (F = 352.18, *p* < 0.001), suggesting that there are substantial differences in the dependent variable between the two levels of VAS. The effect size associated with VAS is substantial (ω^2^ = 0.74). No significant interaction is found between VAS and goniometer use (F = 0.11, *p* = 0.79).

## 4. Discussion

Regarding plantar pressures, our results showed a statistically significant decrease after minimally invasive surgery. Several authors [30,31,32] have performed simulation studies of plantar pressures during statics and dynamics with cadaveric models after performing Weil or Chevron osteotomies on the second metatarsal, finding a significant decrease in plantar pressure under the operated head and increase in the adjacent ones. Although these are studies with limitations due to the number of specimens used and the difficulty in simulating all the phases of gait in a cadaveric model.

Transfer of the overload to adjacent metatarsals is another common complication reported by several authors, Malhotra [33] reports 8%, Biz [34] found 3.2% in his case series, Haque [35] found 3% while de Prado [14,36] and Henry [37] 2.4% and Magnan [38] 2.9%. This complication was not found in our investigation, perhaps because we only operated on the metatarsal affected by primary metatarsalgia.

The findings of this study regarding the use of the inclinometer, are in line with the recommendations of Olivier Laffenêtre, where the importance of performing the metatarsal osteotomy at a 45-degree angle is emphasized [17,39].

Another relevant aspect pointed out by other authors, with which we agree, is that minimally incisional osteotomies (DICMO) cause less edema in the post-operative period due to the inherent stability of the osteotomy being intracapsular, compared to distal minimally invasive osteotomies (DMMO).

When comparing our findings with the scarce literature available on plantar pressures after minimally invasive surgery, a study stands out [40] where pressures were measured after surgery compared with healthy controls, but without pre-operative measurements. On the other hand, Harris et al. [41] did take pre-operative and post-operative measurements after open osteotomies, finding decreased pressures under the operated head, but increased pressures in adjacent heads. Our results provide additional evidence, showing an overall reduction in pressures after minimally invasive surgery, without overloading adjacent radii and without post-operative complications perhaps derived from intra-articular (DICMO).

The AOFAS scale has proven to be a valid and widely used instrument for assessment in foot and ankle surgeries [27,42,43]. Specifically in minimally invasive surgery, several previous studies have used the AOFAS to assess clinical improvement after surgery [44]. In our study, the AOFAS-MTP-IP score reached 92.77 after minimally invasive surgery, approaching normality in individuals without pathology and exceeding the values achieved in other studies on this surgical technique [14,40,45].

Another article prospectively studying the treatment of primary metatarsalgia by minimally invasive surgery [46] found significant improvement of the VAS scale with a decrease from 7.7 ± 1.0 in the initial score to a value of 0.3 ± 0.9 12 months after the intervention.

The VAS scale has been shown to be a useful tool for the subjective assessment of pain in patients with metatarsalgia [47]. This highlights the greater efficacy of minimally invasive surgery in relieving metatarsal pain compared to conservative measures. Our results are in agreement with other studies that evaluated minimally invasive metatarsal osteotomies and also found remarkable decreases in the VAS scale after surgery [33,34,35].

One of the significant limitations of this observational study is the disparity in sample size between patients who underwent osteotomy angle measurement using a digital goniometer and those who did not. This disparity introduces a potential bias in the evaluation of the results, as the total sample is reduced to 50 patients instead of the original 65. The use of the goniometer in only a fraction of the sample, although it may provide relevant information for future research or serve as a pilot study, makes it difficult to generalize the results to the entire population. In addition, the focus is on the secondary objective of osteotomy angle assessment, which may divert attention from the primary objective of the study, which is to assess the change in plantar pressures associated with intracapsular ostomy. Therefore, it is essential to take this bias into account when interpreting the results and to recognize the need for further research with more representative samples and more balanced approaches to fully address the study objectives.

## 5. Conclusions

In conclusion, minimally invasive metatarsal osteotomy using the DICMO technique is a safe and effective treatment for primary metatarsalgia, achieving plantar pressure reduction, improved metatarsal function/alignment, and significant pain relief.

## Figures and Tables

**Table 1:** Sample characteristics.

Variable	All Participants (n= 65)	Non Goniometer (n = 50)	Goniometer (n = 15)	*p*-Value
Age (mean ± sd)	54.69 ± 13.75	55.06 ± 13.63	53.47 ± 14.57	0.70
Height (m)	1.64 ± 0.09	1.64 ± 0.09	1.63 ± 0.08	0.64
Weight (Kg)	66.58 ± 10.22	66.28 ± 10.49	67.57 ± 9.56	0.67
Body mass index (kg/m^2^)	24.85 ± 3.60	24.62 ± 3.44	25.60 ± 4.10	0.36
Sex, n (%)				
Men	17 (26.15%)	14 (21.54%)	3 (4.62%)	0.54
Women	48 (73.85%)	36 (55.38%)	12 (18.46%)	
Laterality, n (%)				
Right	38 (58.46%)	28 (43.08%)	10 (15.38%)	0.46
Left	27 (41.54%)	22 (33.85%)	5 (7.69%)	
Diabetes	8 (12.3%)	6 (12%)	2 (13%)	0.89
Smoking	6 (9.23%)	5 (10%)	1 (6.67)	0.70

**Table 2:** Descriptive data (Mean and SD).

	All Participants (n= 65)	Non Goniometer (n = 50)	Goniometer (n = 15)
Pressure Previous (g/cm)	747.63 ± 215.13	766.68 ± 237.68	684.13 ± 90.73
Pressure 6 months (g/cm)	601.86 ± 208.06	645.58 ± 216.09	456.13 ± 69.80
Pressure 12 months (g/cm)	602.83 ± 206.20	645.66 ± 214.36	460.07 ± 71.81

**Table 3:** Post hoc comparisons of the Goniometer/AOFAS-MTP-IP.

	Time	Mean	ET	Difference	t	Cohen’s d	*p*-Value
Group SI	Pre	-	-	-	-	-	-
	6 months	−42.96	2.06	−42.96	−20.85	−3.72	<0.001
	12 months	−49.00	2.06	−49.00	−23.78	−4.24	<0.001
Group No	Pre	−2.31	3.40	−2.31	−0.68	−0.20	0.98
	6 months	−43.78	3.40	−41.47	−12.87	−3.79	<0.001
	12 months	−51.38	3.40	−49.07	−15.11	−4.45	<0.001

Note. Adjusted *p*-value to compare a family of 15.

## Data Availability

The data presented in this study are available upon request to the corresponding author.
